# Scientific communication and vaccine hesitation: an analysis of the editorial line of a great Brazilian newspaper

**DOI:** 10.1093/oxfimm/iqaf012

**Published:** 2026-03-02

**Authors:** Heslley Machado Silva

**Affiliations:** Science, Health and Education Department, State University of Minas Gerais (UEMG) and University Center of Formiga (UNIFORMG) Cel. Francisco Manuel Franco Street, 175, Centro, Itaúna, Minas Gerais, 35680-053, Brazil

**Keywords:** vaccine hesitation, disinformation, newspaper, childhood vaccination, scientific communication, misinformation

## Abstract

The text critically examines the anti-vaccine editorial line adopted by “Gazeta do Povo”, one of Brazil’s leading newspapers, and its possible repercussions on vaccine hesitancy in the country. By analyzing headlines published over the course of a year, a trend of misinformation and sensationalism was found, addressing supposed or insignificant risks in relation to COVID-19 vaccines. A structured qualitative document analysis was conducted, based on the systematic retrieval of all vaccine-related headlines published by the newspaper between December 2022 and December 2023. Headlines were collected using predefined search terms relevant to COVID-19 vaccination and assessed according to their scientific accuracy and potential to induce vaccine hesitancy, following WHO and CDC communication guidelines. The analysis highlights how such news, often contradictory to scientific knowledge, can negatively influence the decision-making of citizens concerned about their health and that of their children. The article also highlights the responsibility of the press in disseminating reliable information, as well as the need for effective reactions to the anti-vaccine movement and scientific denialism in Brazil and globally.

Many Brazilian parents seeking trustworthy vaccine information, concerned about your own health and that of your children, immersed in a virtual environment of fake scientific news via social media [1] and seeking information from a reliable source that provides them with clarification on the safety of vaccines for themselves and their children. They subscribe to the Gazeta do Povo newspaper, one of the 10 most widely circulated newspapers in the country, which was the most read newspaper in Brazil in the 2018 elections, so it must have credibility. These parents put into the Google search engine the terms: “*Gazeta do povo vacina Covid-19*”. To their surprise, or not, they find the following photo of a nurse from a horror movie with a menacing syringe in her hand.

**Figure:**
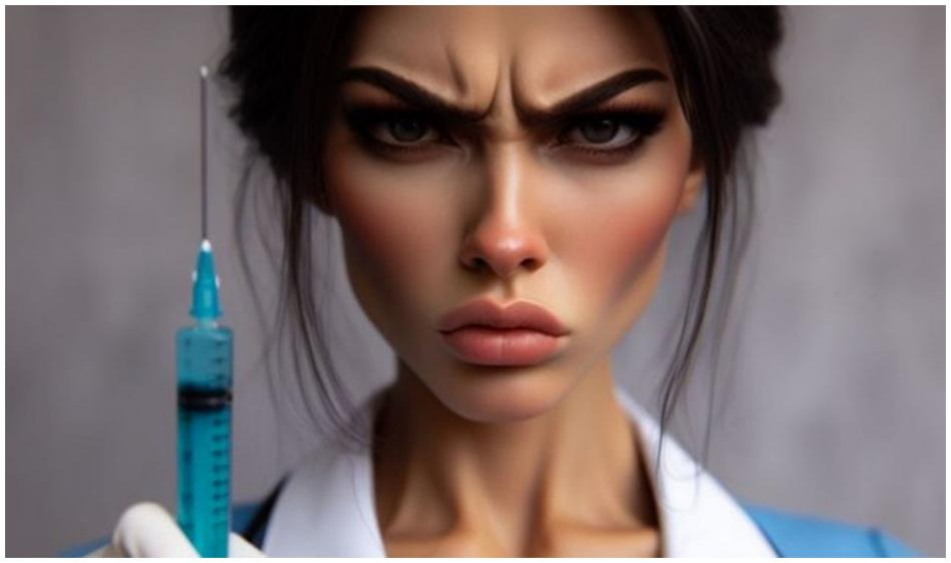


The image (Picture taken from the website of Brazil’s national newspaper Gazeta do Povo (accessed on January 25th, 2024, website: https://www.gazetadopovo.com.br/tudo-sobre/vacina-covid-19/) illustrating the headline: “Nothing to link with science: compulsory vaccination for children is pure ignorance.” November 10th, 2023.) (mis)informs a lot about the supposed risks of vaccination, but the headline (mis)informs a lot more, with the following sentence: “**Nothing to link to science: compulsory vaccination for children is pure ignorance.**” Forgetting for a moment the issue of COVID-19 vaccines, the headline is a general one against childhood immunization. The headline and the image of the article speak against vaccination in a country that was once one of the leaders in immunization in the world, which has seen the return of eradicated and very serious diseases, often deadly, such as measles and the decline of polio vaccination (with the risk of its return), among others, in children. Would these parents have any doubts about giving up vaccinating their children after seeing this photo and reading this headline?

## Methods

A qualitative documentary analysis was conducted to examine how *Gazeta do Povo* framed information related to COVID-19 vaccination. Headlines were selected through a systematic Google search using the terms “Gazeta do Povo vacina COVID-19”, “vacinação”, “dose bivalente”, “AstraZeneca”, “compulsória”, “vacina de RNA mensageiro”, “risco” and “crianças”. The search covered all content published between December 2022 and December 2023.

All retrieved headlines were screened. Four were selected for in-depth analysis because they:

- explicitly referred to alleged vaccine risks;
- contradicted or misrepresented consolidated evidence from WHO, CDC, EMA, and ANVISA;
- used sensationalist language capable of influencing vaccine decision-making.

Headlines were analyzed following WHO’s *Guidelines for Risk Communication and Community Engagement* [2, 3] and CDC’s *Principles of Effective Vaccine Communication* [4, 5]. No human participants were involved; therefore, no ethics approval was required.

## Main text

This analysis occurs within a broader national context in which the Brazilian immunization system has suffered a marked decline in childhood vaccine coverage since 2016, influenced by the political climate, erosion of trust in public institutions, and the circulation of misinformation across medical, religious, and digital communities. According to the Ministry of Health and ANVISA, misinformation became one of the central drivers of falling vaccination rates in the post-pandemic period, amplifying uncertainty among parents and contributing to outbreaks of diseases previously under control, such as measles.

But these parents are very cautious, and continue to trust this newspaper, after all, if it is publishing, there is a journalist who signs it, it has responsible editors and the newspaper would not be irresponsible to publish something that was unfounded [6]. So they decided to look at the headlines about vaccination in this newspaper over the course of a year to find out for themselves on the same web page, as this could be an isolated piece of news discouraging immunization.

The pattern of headlines recurrently emphasizes alleged vaccine risks, potentially reinforcing pre-existing doubts among readers and contributing to a climate of distrust toward immunization. The parents listed the following headlines over the course of a year: end of 2022 to end of 2023:

Headline: “US approves more doses of Covid vaccine, but even expert say they won’t take it.” September 14, 2023.

Headline: “AstraZeneca Covid Vaccine Increased Heart Attack in Young Women 3.5 Times, Scientists Conclude.” March 30, 2023

Headline: “Bivalent Covid dose could be associated with ‘stroke’ risk, CDC and FDA report.” January 18, 2023

Headline: “European Union records more than a thousand cases of vaccine myocarditis in children.” December 18, 2022

There were other headlines along the lines of denying the value of vaccines, which misinform about non-existent or insignificant risks compared to the benefits of immunizers, but these four are enough evidence of the newspaper’s anti-vaccine editorial line. Let’s leave aside those that disseminate innocuous and dangerous drugs against COVID-19, such as chloroquine [7], there were several, but we will focus on the serious issue of immunization and the headlines against the vaccine in this newspaper.

The first selected headline is incisive, “Even an expert says he won’t take it…” it is not the newspaper that is telling the couple not to get vaccinated, or to vaccinate their children, it is an authority on the subject who is saying this, so there should be no doubt about the refusal of the new immunizers. The headline does not contextualize the consensus established by ANVISA, WHO, and global scientific bodies regarding the safety and effectiveness of continued COVID-19 vaccination. There is a clear consensus within the scientific community on the relevance of continued vaccination against SARS-CoV-2. ANVISA (Brazilian health surveillance), WHO (World Health Organization), FDA (Food and Drug Administration) and CDC (Centers for Disease Control and Prevention), among other agencies, the world’s most prominent researchers, all agree that it was mass and continuous immunization that got us out of the pandemic nightmare, but the Brazilian newspaper has found an expert to the contrary and highlights it in its headline [8–14]. And unwary parents are influenced in this way, after all they trust the publication, the supposed authority on the subject, and the headline, they probably will not take any additional doses against COVID-19, they are backed by a national newspaper and an expert.

The second headline is more specific, and perhaps even more harmful in terms of misinforming and scaring the Brazilian population, as it links to a supposedly false generalist conclusion by “scientists”. Heart attack is the leading cause of death in the world, and taking AstraZeneca’s much-publicized, much-used, much-prized, much-safe vaccine would have increased the risk of heart attack among young women by 3.5 times, a typical piece of scientific Fake News. This couple’s teenage daughter has just been denied one of the most interesting ways of being immunized against COVID-19, it is important to point out that it has already been proven that infection with SARS-CoV-2, especially in the form called Covid Long, or simply having had the disease, is a risk factor for heart problems [15, 16], but this kind of information is of no interest to this newspaper and its editors. Imagine if this headline were true, let’s remember that this was one of the most widely used vaccines in the world, impacting three and a half times the leading cause of death in the world, we would have crowded hospitals and cemeteries alike, it would be much worse than the pandemic itself, but we don’t have to look for rationality in these headlines. Such framing may unintentionally amplify misconceptions among the lay public, especially when the scientific evidence is not adequately presented.

The third headline is just as alarming, and just as illogical, but it adds another element of disinformation by linking fake news to two of the most important health agencies in the world. The FDA (Food and Drug Administration) and the CDC (Centers for Disease Control and Prevention) are the benchmarks of the world, and the discourse of these two agencies has always been in favor of vaccination, and they take care of the safety of public health policies in the US and are the benchmark for the world. Now imagine if these two agencies, with their technicians among the best prepared on the planet, had the slightest suspicion that a particular vaccine, in one of its doses, was increasing the risk of “stroke”, one of the most lethal, most incapacitating, most feared diseases that humanity faces. Immediately the bivalent dose of that immunizer would be suspended until all the safety tests had been carried out and this risk had been confirmed or not. The truth is exactly the opposite of what this headline suggests. Once again, it is COVID-19 itself, and its more pernicious form, “long COVID”, that leaves sequelae in the cardiovascular system, increasing the number of stroke cases in the post-pandemic period [17]. But the FDA and the CDC would be pointing out exactly the opposite, nothing more false, but the message is given to our couple, and as they suffer from high blood pressure, something with a high incidence in Brazil [18], it is clear that both of them will give up taking the bivalent booster dose, which would be so important for them. From this (mis)information, something already strongly detected by the Brazilian health system will be encouraged, which is the decline in acceptance of the bivalent vaccine [19].

The latest headline helps parents to decide that they won’t vaccinate their young children. After all, according to the news, more than a thousand children in Europe have died of myocarditis because of a particular vaccine. We would envision the irresponsibility of the European health agency and its governments in knowing, or at least suspecting, which vaccines were causing this tragedy among their children and still continuing to immunize. It’s clear that this approach is unfounded, but it is important to note that we started this analysis from a report that drew attention to the risk of children being vaccinated, and a year earlier, the same newspaper had already taken this anti-vaccine line. Research has obviously shown the opposite: those who have been vaccinated against COVID-19, whether they are adults, the elderly, young people or children, run a much lower risk of suffering from myocarditis [20, 21]. It is a shame for this couple, for their family and for all the readers of this newspaper, because in fact the proven risk of myocarditis is precisely because of the COVID-19 infection [22, 23], that could be avoided or mitigated by immunization, imagine the number of deaths that could result from this kind of misinformation.

Since there has been no mobilization from academia, the health sector, the Public Prosecutor’s Office or the new Brazilian government against these headlines, it must be because this information is true. In the parallel reality of conspiracy theories, other media outlets are thought to be hiding the truth, as are all the health agencies in Brazil, the US and Europe, and the scientists who study and publish on the subject. A typical script for social networks that spread disinformation. So we have a major victory for the anti-vaccine movement in Brazil.

Misleading or decontextualized vaccine coverage has concrete implications for public health. In Brazil, falling childhood immunization rates have contributed to the resurgence of measles outbreaks, declining polio protection, and increased vulnerability to COVID-19 reinfections. Studies from the Ministry of Health indicate that exposure to alarmist or inaccurate media narratives is associated with decreased trust in immunization campaigns and lower vaccine uptake, particularly among parents of young children. Thus, the editorial framing adopted by widely read newspapers constitutes a relevant factor in shaping population-level health behavior.

It is not possible to attribute the decline in vaccination in Brazil to this newspaper alone, after all, we had a government for four years that spread this kind of misinformation, medical organizations (and many doctors) that endorsed this denialism and the ubiquitous social networks that also spread these conspiracy theories. But if you follow the editorial line [24], and when you consider how far-reaching this newspaper is in Brazil, it’s clear that it participates in this growing and intense process of Brazilian vaccine hesitancy.

## Limitations

This analysis is based exclusively on publicly available headlines and does not include the full articles or internal editorial processes. It also does not quantify the effect of media exposure on individual behavior. The aim is not to establish causality but to document patterns of communication that may contribute to a broader ecosystem of vaccine hesitancy. Future studies could adopt mixed-methods approaches, audience surveys, and longitudinal monitoring of misinformation impact.

## Conclusion

The examination of one year of vaccine-related headlines reveals a recurrent pattern of risk-focused, decontextualized, or alarmist framing capable of reinforcing public doubts about COVID-19 immunization. While these headlines operate within a broader environment shaped by political polarization, disinformation on social media, and medical controversies, their potential influence warrants careful attention. Strengthening responsible scientific communication, ensuring consistent alignment with WHO, CDC, and ANVISA guidelines, and promoting media literacy remain essential strategies to counteract vaccine hesitancy in Brazil. The findings presented here aim to contribute to this broader effort.
