# Correction to: Scientific communication and vaccine hesitation: an analysis of the editorial line of a great Brazilian newspaper

**DOI:** 10.1093/oxfimm/iqag006

**Published:** 2026-03-26

This is a correction to: Heslley Machado Silva, Scientific communication and vaccine hesitation: an analysis of the editorial line of a great Brazilian newspaper, Oxford Open Immunology, Volume 7, Issue 1, 2026, iqaf012, https://doi.org/10.1093/oxfimm/iqaf012

In the section Main text, text in the seventh paragraph is emended to read: "The truth is exactly the opposite of what this headline suggests. Once again, it is COVID-19 itself, and its more pernicious form, “long COVID”, that leaves sequelae in the cardiovascular system, increasing the number of stroke cases in the post-pandemic period[…]" instead of: "A verdade é exatamente oposta ao que sugere esta manchete. Novamente, é a própria COVID-19 e sua versão mais perniciosa “Covid longa”, que deixa sequelas no sistema cardiovascular, elevando o número de casos de “derrames” no pós pandemia[…]".

